# Clinical myths in dental bleaching: an evidence-based structured narrative review

**DOI:** 10.1590/1807-3107bor-2026.vol40suppl1062

**Published:** 2026-08-21

**Authors:** Alessandra REIS, Bianca Medeiros MARAN, Deisy Cristina Ferreira CORDEIRO, Laryssa Mylenna Madruga BARBOSA, Renata Maria Oleniki TERRA, Alessandro Dourado LOGUERCIO, Michael Willian FAVORETO

**Affiliations:** (a) Universidade Estadual de Ponta Grossa - UEPG, Department of Restorative Dentistry, Ponta Grossa, PR, Brazil; (b) Universidade Estadual de Ponta Grossa - UEPG, Department of Restorative Dentistry, Ponta Grossa, PR, Brazil

**Keywords:** Tooth Bleaching, Evidence-Based Dentistry, Evidence-Based Practice, In Vitro Techniques, Research Design

## Abstract

This narrative review critically examines common myths in dental bleaching that are often presented as strategies to improve efficacy or reduce adverse effects, despite limited, inconsistent, or context-dependent clinical support. The review integrates evidence from clinical trials and systematic reviews identified through searches, with priority given to higher-level clinical evidence when available. In addition, concepts from Evidence-Based Practice and Cognitive Psychology are used to contextualize why weakly supported protocols may persist in clinical decision-making. The analysis addressed frequently disseminated beliefs in clinical practice and led to the following conclusions: a) dietary restriction does not improve bleaching outcomes; b) preventive use of analgesics and/or anti-inflammatory drugs does not reduce tooth sensitivity (TS); c) higher peroxide concentrations may accelerate early bleaching but increase adverse effects and do not enhance final efficacy; d) prolonged gel exposure beyond recommended times does not produce perceptible additional color change and may increase TS; e) tray scalloping does not consistently reduce patient-perceived gingival irritation; f) increasing gel volume or contact area does not improve final bleaching efficacy and may increase biological cost; g) light-activation does not provide additional clinical benefit; h) gel agitation does not enhance bleaching outcomes; i) over-the-counter products do not replace professionally supervised bleaching in terms of predictability and magnitude of color change; and j) desensitizing toothpastes show inconsistent efficacy in preventing TS. Overall, bleaching protocols should be selected according to clinically meaningful benefits, adverse effects, patient-centered outcomes, and the strength and consistency of the available evidence, rather than tradition, intuition, or commercial appeal.

## Introduction

Dental bleaching is often discussed in terms of color improvement, but color change alone does not fully define clinical success. In an elective esthetic treatment, patients also perceive the experience and value of treatment through outcomes such as tooth sensitivity (TS), gingival irritation (GI), comfort, satisfaction, and improvement in quality oflife.^1^ For this reason, the evaluation of bleaching protocols should go beyond whitening efficacy and include outcomes that reflect how treatment is experienced by patients.

For vital teeth, at-home bleaching, in-office bleaching and their association are the main bleaching protocols supervised by the dentist. Although they are all effective,^1^ they differ substantially in peroxide concentration, application mode, treatment duration, professional involvement, and patient participation.^2^,^3^ These differences may influence not only the magnitude of color change, but also adherence, convenience, adverse effects, costs, treatment time and patient preferences.

Over the past decades, dental bleaching has been extensively evaluated.^1^,^4^ Laboratory and clinical studies have improved the understanding of its mechanisms of action, efficacy, safety, adverse effects, color stability, and patient perception.^1^,^4^ Despite this growing body of clinical evidence, clinical practice is still influenced by old beliefs and recommendations that are often repeated as universal truths, even when the supporting evidence is lacking, limited, inconsistent, or context dependent. Such beliefs commonly arise from past personal experience, training traditions, clinical reports, or inappropriate extrapolation of laboratory findings to clinical practice.

This issue is particularly relevant in dental bleaching because clinicians must make decisions not only about efficacy, but also about safety issues, adverse effects, and patient's related factors. In this context, the application of the principles of evidence-based practice (EBP)^5^,^6^,^7^ provides an appropriate framework to help clinicians integrate the best available evidence with clinical judgment and patient values and preferences.

This structured narrative review aims to critically examine common clinical recommendations in dental bleaching that are frequently presented as strategies to improve efficacy, reduce adverse effects, or simplify treatment protocols. For clarity, these recommendations are referred to as clinical myths when their routine use is not supported by consistent or clinically meaningful evidence. Before addressing each myth, the review briefly contextualizes how biological plausibility, clinical tradition, commercial dissemination, selective interpretation of evidence, or cognitive biases may contribute to the persistence of weakly supported protocols in clinical practice.

### Why weakly supported bleaching protocols persist in clinical practice

The persistence of clinical myths cannot be explained solely by limited access to scientific information. Human cognition is not neutral when processing evidence. In clinical decision-making, evidence is interpreted under uncertainty, and this process may be influenced by cognitive shortcuts.^8^,^9^,^10^ Confirmation bias may favor information that supports previously held beliefs, whereas contradictory findings may be judged more critically or dismissed as exceptions.^11^ Availability bias may give disproportionate weight to personal experiences or memorable cases, and anchoring bias^12^ may cause early learned concepts to continue influencing clinical judgment even after new evidence becomes available.^9^

These mechanisms do not imply lack of knowledge or bad faith. Rather, they illustrate why clinical recommendations may remain familiar and persuasive even when the supporting evidence is limited, inconsistent, or not clinically meaningful, distorting medical decision-making.^13^,^14^

Additionally, clinicians are not always adequately trained during graduate education to understand that different sources of information occupy different positions in the hierarchy of evidence, and that this should be considered when making treatment decisions. EBP^6^ does not diminish clinical experience. Instead, it helps calibrate clinical judgment by integrating the best available scientific evidence with professional expertise and patient values, needs, and expectations.^15^

In bleaching treatment, this integration is particularly important because protocols are often selected in situations that involve biological variability, esthetic expectations, adverse effects, cost, treatment time, and patient comfort. Therefore, clinicians should assess not only whether a protocol is biologically plausible or commercially available, but whether it has demonstrated clinically meaningful benefits that outweigh its potential harms. This is especially relevant when attractive claims are adopted before being adequately supported by methodologically sound clinical evidence.

Social media may intensify this problem by favoring visual appeal, simplification, and confidence over scientific credibility.^15^ This is especially problematic because protocols may become popular before being properly validated, immediate visual effects may be confused with true bleaching, and persuasive language may replace biological rationale, clinical follow-up, and comparison with well-established techniques. In this environment, what appears convincing may be mistaken for what is scientifically supported.^15^

A common example is the promise of "bleaching with zero TS." This claim is questionable because it presents a relative benefit as if it were an absolute outcome. The best available strategy to reduce bleaching-related TS is the use of a potassium nitrate desensitizer.^16^ Although this approach may reduce the risk and intensity of TS, the available evidence does not support complete prevention. Its benefit is limited, with about one in every eight treated patients avoiding TS because of the intervention itself (number needed to treat = 8).^16^

Another recurrent source of misinformation involves "natural," "vegan," or "peroxide-free" products, such as activated charcoal, violet toners, color-correcting serums, and extract-based formulations.^17^,^18^,^19^ The key question in these cases is whether there is a biologically plausible mechanism capable of producing true bleaching. Since the main mechanism of dental bleaching is the oxidation of organic molecules,^20^ products that do not act through this pathway are more likely to produce superficial stain removal, abrasion, or temporary optical masking rather than true bleaching.^17^ Therefore, describing them as whiteners in the same biological sense as peroxide-based agents is inaccurate.

## Methodological approach

This article was designed as a structured narrative review. The methodological reporting was guided by the SANRA scale for the quality assessment of narrative review articles.^21^ The objective was not to conduct a systematic review, scoping review, or meta-analysis, but to critically synthesize and contextualize clinical evidence related to common myths in dental bleaching that remain frequently disseminated in clinical practice.

A broad literature search was performed in MEDLINE via PubMed, Embase, and the Cochrane Library for each myth described in this article. The search strategy included descriptors and free terms (MeSH and Emtree), related to bleaching, whitening, hydrogen peroxide (HP), carbamide peroxide (CP), TS, GI, along with specific filters to identify randomized controlled trials (RCTs), clinical trials, and systematic literature reviews addressing each one of the myths herein discussed. Additional terms were adapted for each clinical myth, such as diet, analgesics, anti-inflammatory drugs, application time, bleaching tray design, gel volume, light activation, violet LED, gel agitation, over-the-counter products, toothpastes, and desensitizing agents.

Because this was a narrative review, strict predefined eligibility criteria, data extraction and quantitative synthesis were not applied. Priority was given to systematic reviews, randomized controlled clinical and non-RCTs addressing the following clinically relevant outcomes: color change, TS, GI, patient-reported outcomes, and safety. Laboratory and mechanistic studies were considered when they helped explain the biological plausibility of any myth, but conclusions were primarily based on clinical evidence whenever available. When systematic reviews were available, we did not cite the primary articles addressed in the systematic review to avoid overcitation.

To improve transparency and reduce the risk of selective interpretation, the risk of bias of the studies cited was assessed using specific tools: RoB 2.0 for RCTs,^22^ ROBINS-I for non-RCT studies,^23^ and AMSTAR 2 for systematic reviews.^24^ These assessments were used not as a basis for quantitative grading, given that this is not a systematic review, but to support the interpretation of the strength and limitations of the evidence discussed throughout the review.

### Debunking myths in dental bleaching

**Figure 1:**
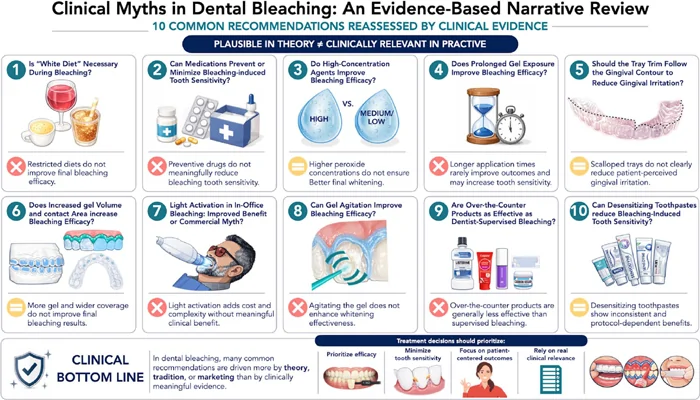
Graphical summary of ten common clinical myths in dental bleaching and their evidence-based reassessment.

The following sections discuss ten clinical recommendations commonly disseminated in dental bleaching and examine whether they are supported by consistent, clinically meaningful, and methodologically reliable evidence (Figure 1).

### Is "White Diet" necessary during bleaching?

The recommendation for patients to follow a "white diet" during dental bleaching procedures originated from the assumption that bleaching agents could temporarily alter the enamel microstructure,^20^ making it more susceptible to pigment adherence. Laboratory findings^25^,^26^ showed that peroxide-based gels might increase enamel surface roughness and porosity, which could theoretically facilitate stain absorption from chromogenic substances such as coffee, tea, or wine.

Although these in vitro studies suggest some biological plausibility, this effect does not appear to translate into clinically meaningful outcomes. In situ studies have not supported this assumption,^27^,^28^ likely because saliva plays an important role in reversing enamel surface changes, such as roughness and porosity. In addition, controlled clinical trials have consistently shown that the consumption of pigmented beverages, such as coffee,^29^ cola-based soft drinks,^30^ tea and coffee,^31^ and red wine^32^,^33^ does not significantly impair bleaching efficacy as confirmed in recent systematic reviews of the literature.^34^,^35^

**Figure 2:**
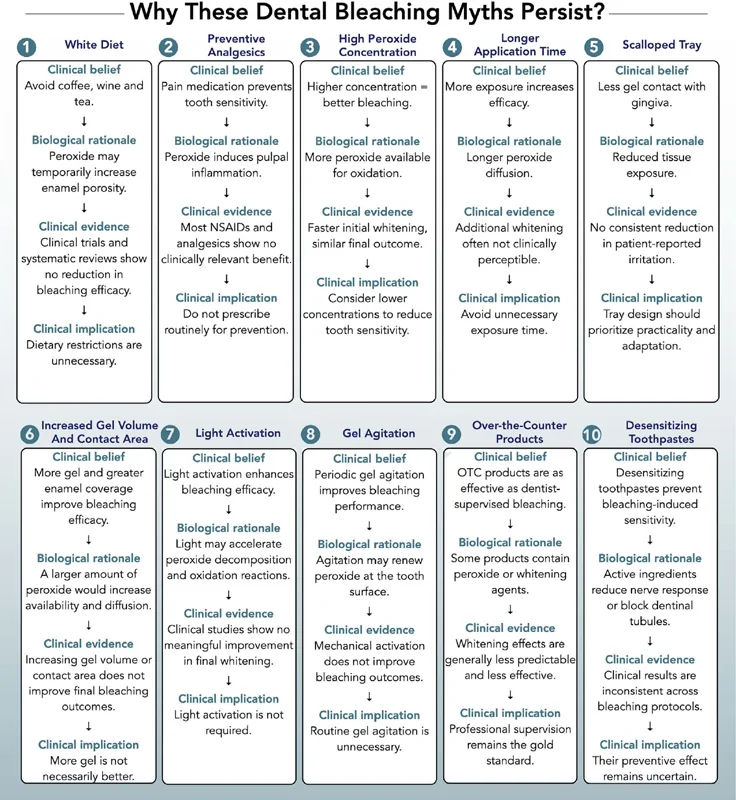
Summary of the current evidence on the ten common myths in dental bleaching.

From a clinical perspective, dietary restriction during bleaching is not supported by the available clinical evidence. Although laboratory studies provide biological plausibility for transient enamel surface changes after peroxide exposure, controlled clinical studies and systematic reviews consistently indicate that consumption of colored beverages does not impair bleaching efficacy. Therefore, recommending a "white diet" may unnecessarily restrict patients without providing a measurable clinical benefit (Figure 2).

### Can medications prevent or minimize bleaching-induced tooth sensitivity?

The rationale for prescribing analgesics or anti-inflammatory drugs before in-office dental bleaching^36^,^37^,^38^ is based on the fact that HP, when reaching the pulp tissue, triggers an acute inflammatory response in the dental pulp.^37^,^38^ Thus, using preventive nonsteroidal anti-inflammatories (NSAIDs) or analgesics could be a way to reduce the release of inflammatory mediators such as prostaglandins, lessening bleaching-induced TS and improving patient comfort.^36^,^37^,^38^

However, the available evidence has not supported this theoretical expectation.^36^,^37^,^38^ Orally administered drugs studied to date, including ibuprofen, dexamethasone, naproxen, etoricoxib, acetaminophen/paracetamol, dipyrone/metamizole, and lornoxicam, have not been effective in reducing bleaching-related TS, as confirmed by systematic reviews of the literature.^36^,^37^,^38^ Although a slight immediate reduction in TS has been reported,^38^ the magnitude of this effect is not clinically relevant. These findings may be explained, at least in part, by the involvement of mediators not inhibited by conventional NSAIDs, as well as by the limited penetration of systemically administered drugs into pulpal tissues.^36^,^37^

In addition to systemic approaches, topical application of pharmacological agents has also been investigated and was the subject of a recent systematic review with meta-analysis.^39^

The pooled analysis of agents such as otosporin, eugenol, ibuprofen combined with arginine, and dipyrone showed no consistent benefit in reducing TS during in-office bleaching.^39^ Among these approaches, ibuprofen combined with arginine showed the most promising result.^40^ However, there is only a single RCT, limiting generalizability, and no commercial product is currently available. Therefore, this combination still deserves further evaluation.

From a clinical perspective, patients should be informed that bleaching-induced TS is a common but usually transient adverse effect, generally resolving within 24 to 48 hours.^41^ Current evidence does not support the routine preventive prescription of systemic or topical analgesic and anti-inflammatory drugs to avoid bleaching-induced TS. This conclusion is supported by systematic reviews showing no clinically meaningful reduction in TS for most tested agents, although isolated formulations, such as ibuprofen combined with arginine, still require further clinical validation (Figure 2). Therefore, medications should not be presented to patients as an evidence-based strategy to prevent bleaching-induced TS. If pain occurs during or after treatment, pharmacological management may be considered only as symptomatic care, according to general clinical principles, patient medical history, contraindications, and professional judgment.

### Do high-concentration agents improve bleaching efficacy?

The wide variety of bleaching products makes product selection challenging for clinicians. Because peroxide concentration is one of the most visible product characteristics, it often drives the assumption that higher concentrations produce greater bleaching efficacy. This reflects the intuitive but oversimplified idea that more active agent means better results.

Although this heuristic may seem reasonable, it is not a reliable basis for decision-making in a complex biological system. In at-home bleaching, some RCTs^42^,^43^ showed that higher CP concentrations may produce greater color change during the first days or first week of treatment, but this advantage is time-dependent and diminishes with continued use, as demonstrated in subsequent trials.^43^,^44^,^45^ Aligned with these findings, a systematic review concluded that 10% CP achieves comparable bleaching efficacy to 16% and 22% CP within the usual treatment period.^46^ A similar pattern was observed for HP-based at-home gels, with no significant difference in color change between lower (4% HP) and higher concentrations (10% HP) over a two-week treatment period.^45^

More recent evidence synthesis supports these findings. A network meta-analysis including 99 RCTs^47^,^48^ showed that most at-home bleaching concentrations provide comparable efficacy. Although the overall certainty of evidence in systematic reviews was rated as moderate to low, individual RCTs with low risk of bias consistently reported similar efficacy across higher and lower peroxide concentrations.^45^,^49^,^50^ Comparable findings have also been observed in long-term follow-up studies.^51^,^52^,^53^

These conclusions are not protocol dependent. In-office bleaching traditionally uses 35% HP, but lower concentrations, including low (≤ 20%) and medium (20 to 30%) HP gels, have shown similar bleaching efficacy to high-concentration products (≥ 30%).^54^ This finding is biologically plausible, since a substantial proportion of the active HP, around 70%, may remain on the enamel surface even after a 50-minute application of a 35% gel,^55^ suggesting that lower concentrations may still provide sufficient peroxide availability to achieve comparable outcomes. Despite this evidence, low-concentration in-office products remain uncommon in the market.

Another important finding across these studies, in addition to the similar bleaching efficacy, is the difference in adverse effects. In both at-home and in-office bleaching, lower-concentration gels have been consistently associated with a lower risk and lower intensity of TS than higher-concentration products.^46^,^47^,^48^,^54^

From a clinical perspective, higher peroxide concentration should not be interpreted as synonymous with superior bleaching efficacy. Although higher concentrations may accelerate color change during the early phase of treatment, the available clinical evidence indicates that this initial advantage tends to diminish over time and does not usually translate into superior final whitening within common treatment periods. Therefore, peroxide concentration should be selected according to the balance between expected efficacy and adverse effects, particularly TS. For patients with higher susceptibility to discomfort, such as those with non-carious cervical lesions, enamel crack lines, small teeth, gingival recession, or multiple restorations, lower-concentration gels may be a more appropriate evidence-based option because they can provide comparable final bleaching outcomes with a lower risk or intensity of TS (Figure 2).

### Does prolonged gel exposure improve bleaching efficacy?

For teeth to become lighter, HP must diffuse through the dental structure and oxidize its organic components.^20^ Based on this, longer application times are intuitively assumed to produce greater color change. Some laboratory studies^56^,^57^,^58^,^59^ and a controlled trial^60^ comparing short daily CP applications of15 and 30 min with an 8-hour protocol support this assumption, showing greater whitening with longer contact time. However, this relationship is unlikely to remain linear throughout treatment. As the concentration gradient decreases, peroxide is gradually consumed or degraded, and fewer oxidizable (unsaturated double carbon bonds) substrates remain available. Consequently, extending gel exposure may produce progressively smaller increments in color change, which may no longer be clinically perceptible despite longer application times.

In the trial by Cardoso et al. 2010,^60^ for example, no difference was detected between the 1-hour group treated for 18 days and the 8-hour group treated for 16 days. A comparable trend has also been reported for in-office bleaching protocols requiring gel replenishment, in which color change increased from one 15-min application to three 15-min applications.^61^ More recent controlled trials reinforce this interpretation. A non-inferiority trial 62 showed that daily application of 10% CP for 2 or 4 hours achieved bleaching results comparable to the standard 8-hour protocol. Similarly, another recent in-office bleaching trial showed that a single application of 30 min resulted in a similar bleaching outcome to a 40-min application.^63^

At first sight, these findings may seem inconsistent with a systematic review^64^ indicating that protocols following the manufacturer's recommended application time generally result in better bleaching efficacy than shortened protocols. However, statistical significance alone does not necessarily imply clinical relevance. The pooled differences did not reach the 50:50 acceptability threshold of 2.7 ΔEab,^65^ suggesting that the advantage of longer application times is not perceived clinically. At the same time, prolonged contact time is associated with a greater risk of TS,^63^ likely due to increased peroxide diffusion toward the pulp.^66^ Therefore, extending application time without a corresponding clinically meaningful benefit may not be justified, particularly when it increases adverse effects. This highlights the importance of carefully weighing potential gains in bleaching efficacy against the higher risk of TS when selecting the bleaching protocol.

**From a clinical perspective,** manufacturer-recommended application times are shown to be longer than necessary to achieve similar whitening outcomes. Evidence from at-home and in-office trials suggests that, within the protocols evaluated, shorter application times can often achieve comparable clinically relevant color change while reducing the risk or intensity of bleaching-induced TS. Therefore, application time should be individualized according to the bleaching product, patient sensitivity, expected adherence, and treatment goals. Longer exposure may be justified only when it is consistent with the evaluated protocol and when the expected benefit outweighs the higher risk of discomfort (Figure 2).

### Should the tray trim follow the gingival contour to reduce gingival irritation?

Two tray trim designs are traditionally used for at-home dental bleaching: non-scalloped and scalloped. In the non-scalloped design, the tray extends beyond the gingival margin. In the scalloped design, the tray follows the cervical contour of the teeth and the gingival margin. The scalloped trim is often recommended based on the assumption that it reduces contact between the bleaching gel and the gingival tissue, thereby minimizing the risk of GI. In contrast, the non-scalloped design may provide better gel sealing and is simpler and more reproducible to fabricate.

Current clinical evidence does not support a clear advantage of scalloped trays in reducing patient-perceived GI. A split-mouth RCT^67^ and a parallel RCT^68^ with 120 and 72 participants, respectively, found similar patient-reported risk and intensity of GI. Although one trial reported slightly greater examiner-detected GI with one tray design,^66^ this difference was not accompanied by greater patient-reported discomfort and should therefore be interpreted with caution. In this context, patient-perceived irritation is more clinically relevant than minor examiner-detected changes that do not affect comfort or adherence.^69^

From a clinical perspective, tray scalloping should not be presented as an evidence-based requirement to prevent GI during at-home bleaching. Tray design may instead be selected according to adaptation, retention, gel sealing, ease of fabrication, and patient comfort. When these practical aspects are prioritized, a non-scalloped trim may be a reasonable option because it is simpler to fabricate and may provide better retention and gel containment (Figure 2).

### Does increased gel volume and contact area increase bleaching efficacy?

Intuitively, it may seem that increasing gel volume and enamel contact area would enhance bleaching efficacy by increasing peroxide availability and diffusion. This rationale helps explain practices such as placement of reservoirs in custom bleaching trays or the use of thicker gel layers during in-office bleaching.

In at-home bleaching, increasing gel volume or contact area does not appear to provide a clinically relevant improvement in efficacy. The use of tray reservoirs,^70^ a common strategy to increase gel volume, did not improve color change compared with trays without reservoirs, and no differences were observed in TS or GI.^71^ These findings also remained stable after 1 year of follow-up.^72^ Likewise, expanding the application area to both buccal and palatal surfaces did not accelerate or enhance bleaching efficacy compared with buccal application alone.^73^

A comparable pattern has been observed in in-office bleaching. Esteves et al. 2022,^74^ came closest to evaluating a dose-response relationship by comparing different gel volumes. Slightly greater color changes were observed at some intermediate assessment periods, but the final bleaching outcome was not different across groups. In contrast, TS increased in proportion to the volume applied. Consistently, reducing the thickness of the applied gel did not compromise color change in split-mouth RCTs using either 35% HP^75^ or 6% HP gel^76^ for in-office bleaching.

RCTs demonstrated that full surface coverage may not be necessary to achieve homogeneous and effective bleaching. Restricting gel application to specific buccal areas, such as cervical and incisal regions, was comparable to application to the entire buccal surface.^77^ Likewise, applying the gel only to the middle and incisal thirds also produced similar results to full-surface application.^78^ Homogeneous color change has been reported in patients undergoing bleaching while wearing orthodontic brackets,^79^ highlighting that once HP diffuses into the tooth structure, it may spread three-dimensionally beyond the directly exposed area.

From a clinical perspective, increasing gel volume or contact area should not be assumed to improve final bleaching efficacy. Current clinical evidence indicates that tray reservoirs, thicker gel layers, and expanded application areas do not consistently produce superior final color change when compared with more conservative gel application (Figure 2). This finding is clinically plausible because, after peroxide penetrates the dental structure, diffusion may occur beyond the area of direct surface contact. Therefore, increasing the amount of gel or the treated surface area may mainly increase tissue exposure, treatment cost, and risk of adverse effects without providing a measurable additional bleaching benefit. Gel application should be guided by adequate adaptation, controlled placement, patient comfort, and the minimum amount necessary to achieve predictable contact with the target surface.

### Light activation in in-office bleaching: improved benefit or commercial myth?

Light sources have long been incorporated into in-office bleaching protocols based on the assumption that light energy could accelerate HP decomposition and increase the formation of reactive oxygen species.^80^ This rationale has been investigated with different technologies, including halogen lamps, LED systems, lasers, and, more recently, violet LED devices, used either alone or in association with peroxide-based gels.^81^ However, the chemical plausibility of increased peroxide decomposition does not necessarily imply a clinically meaningful improvement in tooth color, because bleaching outcomes also depend on peroxide concentration, contact time, diffusion through dental tissues, saturation of the bleaching response, baseline tooth color, and patient-related factors. In addition, concerns about temperature increases on the dental surface and within the pulp chamber have raised questions about the balance between potential benefits and harms of light-activated bleaching.^82^

Overall, evidence from RCTs, systematic reviews, and meta-analyses does not support a meaningful benefit of any type of light activation on bleaching efficacy.^83^,^84^,^85^,^86^,^87^,^88^ Across the protocols evaluated, including different light sources and peroxide concentrations, any slight improvement reported in some comparisons was not consistently maintained and did not clearly translate into clinically relevant final color change and may be accompanied by increased TS.^88^ Therefore, the available evidence does not indicate that light activation with any source provides superior whitening compared with bleaching protocols without light.^81^,^89^,^90^

More recently, violet LED has been proposed as an alternative approach, either alone or combined with peroxide-based gels, with claims of effective whitening and lower TS. Although isolated findings have suggested a possible improvement in color change when violet LED was combined with CP,^83^ this evidence is based on only three trials and with limited clinical relevance.^84^ In contrast, RCTs and network meta-analyses have shown that violet LED alone produces less color change than peroxide-based protocols and that its association with peroxide does not provide a meaningful clinical advantage over peroxide alone.^85^,^86^,^87^

From a clinical perspective, light activation should not be considered a necessary component of in-office bleaching. The absence of consistent clinical benefit applies to the light-assisted protocols that have been evaluated to date, including the available visible light sources and violet LED, either alone or combined with peroxide-based gels (Figure 2). This does not mean that every possible combination of wavelength, exposure time, device, and peroxide concentration has been tested. Rather, it means that, in the absence of direct clinical evidence showing superiority for a specific light-assisted protocol, clinicians should prefer bleaching protocols with demonstrated efficacy, safety, and patient-centered benefit. The continued marketing of light-based devices may reflect visual appeal, novelty, technological perception, and consumer demand more than evidence of clinically relevant superiority over conventional peroxide-based bleaching.

### Can gel agitation improve bleaching efficacy?

Agitation of the bleaching gel during in-office application is commonly recommended in some manuals and manufacturers' instructions.^91^,^92^ It is claimed that moving the gel across the dental surface every 5 to 10 min helps release oxygen bubbles and renew the material at the tooth interface,^93^,^94^ thereby enhancing bleaching. The rationale behind this protocol is that the oxygen bubbles released during gel decomposition reduce the continuity of contact between the bleaching agent and the tooth surface. However, this mechanism-based assumption has not been supported by clinical evidence.

RCTs evaluating ultrasonic^93^ or sonic activation^95^,^96^ using high or low active HP concentrations have shown no improvement in color change and no reduction in adverse effects when compared with passive gel application. Because these studies were classified as having low risk of bias, the absence of measurable clinical benefit provides a relatively consistent basis for considering periodic gel movement unnecessary within the protocols evaluated. The interpretation is also biologically plausible since HP is a highly unstable oxidizing agent capable of dissociating into reactive species^91^,^93^,^94^ upon contact with the dental structure^93^,^94^ without requiring mechanical agitation.

From a clinical perspective, manual agitation or vibrating devices should not be considered necessary to improve in-office bleaching efficacy (Figure 2). HP can dissociate and diffuse through dental tissues without mechanical activation, and the available clinical evidence indicates that additional gel movement does not translate into superior whitening or improved patient comfort. Therefore, omitting this step may simplify the clinical procedure without compromising bleaching outcomes.

### Are over-the-counter products as effective as dentist-supervised bleaching?

The growing demand for esthetic dentistry has expanded the market for over-the-counter (OTC) peroxide-based whitening products, including toothpastes, strips, mouthrinses, and paint-on gels.^97^,^98^ These products are often promoted as simple and affordable alternatives to professional bleaching. However, whitening efficacy depends not only on the presence of peroxide, but also on factors such as concentration, contact time, delivery system, and professional supervision.^97^

Among OTC products, whitening toothpastes show heterogeneous mechanisms and limited intrinsic bleaching potential. Most act mainly through abrasives that remove extrinsic stains rather than bleach the tooth structure itself.^99^,^100^ Charcoal-based products have shown effects comparable to regular fluoridated toothpastes, but remain less effective than CP bleaching^101^ and may present disadvantages such as lower acceptability, lack of fluoride, and risk of soft tissue irritation. Any perceived whitening is largely related to superficial stain removal or temporary visual contrast rather than true intrinsic bleaching.^102^ Optical agents such as blue covarine may produce an immediate visual effect^103^,^104^ by depositing a thin semi-transparent film on the enamel, but this is temporary and does not translate into superior intrinsic bleaching over time.^103^,^105^,^106^,^107^,^108^

Other formulations, including enzyme-based (e.g., containing bromelain, papain, or lactoperoxidase) and sodium phytate toothpastes, may improve stain removal or whiteness indices, but these effects are inconsistent or often insufficient to meet patients' esthetic expectations.^19^,^109^,^110^,^111^,^112^ Toothpastes containing low concentrations of HP (0.75% and 2.8%) can produce perceptible color change, but only after prolonged use (12 weeks; 3 times a day) and with lower efficacy than professionally supervised bleaching.^113^ Their main clinical role may be in post-treatment maintenance rather than as a substitute for bleaching therapy, by managing extrinsic stains.^114^

Whitening strips appear to be the most effective OTC option. Although supervised at-home bleaching generally results in greater color change, the difference compared with strips may be small and sometimes clinically imperceptible.^115^ Strips also tend to cause less TS, likely because they use smaller gel volumes and provide more controlled exposure.^115^,^116^ Even so, safety should not be assumed uncritically, because although some studies found no evidence of genotoxic effects,^117^ others reported cellular alterations and oxidative stress markers after strip use.^118^

Other OTC delivery systems, such as paint-on gels and mouthrinses, show more limited efficacy. Paint-on gels lack a physical barrier, which reduces contact time and makes them more vulnerable to removal by saliva and lip movement, resulting in lower efficacy and greater color relapse within 2 weeks to 6 months post-treatment.^119^,^120^ Peroxide-based mouthrinses are even less effective, usually requiring extended use before any relevant color change becomes noticeable, and still producing results inferior to tray-based bleaching.^121^

A recent network meta-analysis provides a clearer hierarchy of whitening efficacy among available protocols. Although several OTC approaches are available, dentist-supervised at-home bleaching with 10% CP (with a total accumulated contact time ≥ 14 h) remains statistically superior to most OTC options, except for 6% HP strips (≥ 14 h), for which no significant difference was observed.^97^ Specifically, 6% HP strips (≥ 14 h) were found to be more effective than 6% HP paint-on gels (7-13 h), which often underperform due to application difficulties reported by users.^97^,^120^ This reinforces the concept that the delivery system and user compliance, factors often managed by professional guidance, are as critical as the active ingredient itself.

From a clinical perspective, OTC whitening products should not be interpreted as a single therapeutic category. Their effects vary according to mechanism of action, peroxide concentration, contact time, delivery system, user compliance, and whether the product produces intrinsic bleaching, extrinsic stain removal, or temporary optical masking. Whitening strips appear to provide the most consistent color change among OTC options because they maintain peroxide in contact with the tooth surface for a defined period, whereas toothpastes, mouthrinses, and paint-on gels are more limited by short contact time, dilution, salivary clearance, or difficulty maintaining the product on the tooth surface (Figure 2). Therefore, although some OTC pro ducts may provide short-term improvement, they should not be considered equivalent substitutes for professionally supervised bleaching in terms of predictability,^97^ magnitude of color change, diagnosis, and follow-up. Their use should be recommended with caution, especially in patients with exposed dentin, gingival recession, non-carious cervical lesions, restorations, or pre-existing sensitivity.

### Can desensitizing toothpastes reduce bleaching-induced tooth sensitivity?

The routine recommendation of desensitizing toothpastes to control TS during bleaching is not consistently supported by clinical evidence, although the evidence is mainly composed of studies with unclear/high risk of bias. This practice is often based on the assumption that products effective against cervical dentin hypersensitivity, through nerve depolarization (e.g., potassium salts) or dentinal tubule occlusion, would also reduce bleaching-induced pain.^122^,^123^ However, the biological rationale is not the same. While dentin hypersensitivity is mainly explained by the hydrodynamic theory, bleaching-induced TS is more consistently understood as a reversible pulpitis caused by the rapid diffusion of peroxide radicals through enamel and dentin, with subsequent activation of pulpal nociceptors and inflammation.^124^,^125^ Therefore, agents that act mainly by occluding dentinal tubules may be mechanistically inadequate for this type of pain.^111^

Clinical evidence is inconsistent and seems to depend on both the active ingredient and the bleaching protocol. Potassium nitrate, which aims to reduce nerve excitability, has shown conflicting results, with some studies reporting reduced TS and others showing no preventive effect during in-office bleaching.^126^,^127^ A possible explanation is that its diffusion toward the pulp is time-dependent, whereas routine brushing may not provide sufficient contact time to counteract the oxidative challenge imposed by high-concentration peroxides.^127^

A similar inconsistency has been observed with tubule-occluding agents such as arginine and bioactive glass. Some studies suggest benefit in at-home bleaching with 22% CP,^128^ whereas others found no reduction in TS during in-office bleaching with high-concentration HP^122^,^123^ even with daily use. In contrast, when bioactive glass was delivered in custom trays for a prolonged period during at-home bleaching with 20% CP, TS was significantly reduced without compromising color change.^129^ This suggests that the method of delivery and exposure time may be as important as the desensitizing agent itself.^129^

Systematic reviews of RCTs reinforce that the effect of desensitizing toothpastes is protocol-dependent. These products may help reduce TS in some situations, such as high-concentration at-home bleaching or single-session in-office bleaching, but they appear ineffective in low-concentration at-home protocols and in multi-session in-office bleaching with high-concentration peroxide.^124^

From a clinical perspective, standard brushing with desensitizing toothpastes should not be considered a reliable evidence-based strategy to prevent bleaching-induced TS (Figure 2). Their limited and inconsistent effect is biologically plausible, because bleaching-induced TS is primarily associated with peroxide diffusion and transient pulpal inflammation, whereas many desensitizing tooth pastes are designed to manage cervical dentin hypersensitivity through nerve depolarization or dentinal tubule occlusion.

These products may still be useful for patients with pre-existing dentin hypersensitivity, and some agents may reduce discomfort under specific bleaching protocols or when delivered with sufficient contact time. However, they should not be regarded as the main preventive approach for bleaching-induced TS. In clinical practice, professional supervision and protocol adjustments, including peroxide concentration, exposure time, application mode, and treatment intervals, remain more consistent strategies to reduce patient discomfort.

## Overall interpretation, strength of evidence, and clinical implications

The evidence reviewed across the ten myths shows that the strength of the conclusions varies according to the type of clinical recommendation, the amount of available evidence, the consistency of findings, and the methodological quality of the studies.^130^ For some topics, such as dietary restriction during bleaching, gel agitation, light activation, peroxide concentration, and application time, the available clinical evidence provides a relatively consistent basis for clinical decision-making, especially when findings from RCTs and systematic reviews converge. In these situations, the absence of clinically meaningful benefit supports the recommendation that these procedures should not be routinely adopted when they increase treatment complexity, cost, patient burden, or adverse effects.

The risk-of-bias assessment (Table) helped distinguish conclusions supported by a more reliable body of evidence from those requiring greater caution. Greater confidence was assigned to topics in which low-risk RCTs, systematic reviews, and meta-analyses with a low-risk of bias predominate in the narrative synthesis, such as myths about white diet (myth 1), preventive medicines to reduce TS (myth 2), the use of high-concentration agents (myth 3), prolonged gel exposure (myth 4), gel volume or contact area (Myth 6), light activated bleaching (myth 7) and gel agitation (Myth 8). Across these topics, the available evidence consistently indicates that protocol intensification, by adding light, mechanically agitating the gel, increasing peroxide concentration, prolonging exposure time, or increasing gel volume or treated surface area, does not usually provide clinically meaningful additional benefit and may increase cost, complexity, treatment burden, or adverse effects.

**Table:** Summary of clinical evidence and methodological appraisal of studies included in the narrative review according to their appropriate assessment tool.

Myth	Study ID	Study design	Appraisal tool	Overall methodological judgment
1. Is "white diet" necessary during bleaching?	Chen et al., 2020^31^	RCT	RoB 2.0	
	Souza et al., 2 0 22^33^	RCT	RoB 2.0	
	Rezende et al., 2013^29^	Non-RCT	ROBINS-I	
	Hass et al., 2019^30^	Non-RCT	ROBINS-I	
	Menezes et al., 2021^32^	Non-RCT	ROBINS-I	
	Hardan et al., 2024^34^	SR/MA	AMSTAR 2	
	Münchow et al., 2 0 2 5^35^	SR/NMA	AMSTAR 2	
2. Can medications prevent or minimize bleaching-induced tooth sensitivity?	Hortkoff et al., 2 0 24^40^	RCT	RoB 2.0	
	Faria-e-Silva et al., 2015^36^	SR/MA	AMSTAR 2	
	Carregosa Santana et al., 20 19^37^	SR/MA	AMSTAR 2	
	Costa et al., 2020^38^	SR/MA	AMSTAR 2	
	Oliveira et al., 2024^39^	SR/MA	AMSTAR 2	
3. Do high-concentration agents improve bleaching efficacy?	Kihn et al., 2000^42^	RCT	RoB 2.0	
	Braun et al., 2007^43^	RCT	RoB 2.0	
	Matias et al., 2000^44^	RCT	RoB 2.0	
	Chemin et al., 2018^45^	RCT	RoB 2.0	
	Lopez Darriba et al., 2017^50^	RCT	RoB 2.0	
	Sutil et al., 2020^49^	RCT	RoB 2.0	
	Tay et al., 2012^51^	RCT	RoB 2.0	
	Meireles et al., 2010^52^	RCT	RoB 2.0	
	Estay et al., 2020^53^	RCT	RoB 2.0	
	Gummy et al., 2024^55^	RCT	RoB 2.0	
	Geus et al., 2018^46^	SR/MA	AMSTAR 2	
	Terra et al., 2025^47^	SR/NMA	AMSTAR 2	
	Terra et al., 2025^48^	SR/NMA	AMSTAR 2	
	Maran et al., 2020^54^	SR/MA	AMSTAR 2	
4. Does prolonged gel exposure improve bleaching efficacy?	Lopez Darriba et al., 2017^50^	RCT	RoB 2.0	
	Cardoso et al., 2010^60^	RCT	RoB 2.0	
	Kose et al., 2016^61^	RCT	RoB 2.0	
	Terra et al., 2025^62^	RCT	RoB 2.0	
	Favoreto et al., 2024^63^	RCT	RoB 2.0	
	Melo et al., 2024^64^	SR/MA	AMSTAR 2	
5. Should the tray trim follow the gingival contour to reduce gingival irritation?	Carneiro et al., 2022^67^	RCT	RoB 2.0	
	Pereira-Lores et al., 2024^68^	RCT	RoB 2.0	
6. Does increased gel volume and contact area increase bleaching efficacy?	Martini et al., 2020, 2021^71,72^	RCT	RoB 2.0	
	Forville et al., 2024^73^	RCT	RoB 2.0	
	Esteves et al., 2022^74^	RCT	RoB 2.0	
	Centenaro et al.^75^	RCT	RoB 2.0	
	Carneiro et al.^76^	RCT	RoB 2.0	
	Esteves et al., 2022^77^	RCT	RoB 2.0	
	Sa et al., 2021^78^	RCT	RoB 2.0	
	Montenegro-Arana et al., 2016^79^	RCT	RoB 2.0	
7. Light activation in in-office bleaching: improved benefit or commercial myth?	Brugnera et al., 2020	RCT	RoB 2.0	
	Zanin et al., 2025a^85^	RCT	RoB 2.0	
	Zanin et al., 2025b^86^	RCT	RoB 2.0	
	Bessa et al., 2025^84^	SR/MA	AMSTAR 2	
	Favoreto et al., 2026^87^	SR/NMA	AMSTAR 2	
	He et al., 2012^88^	SR/MA	AMSTAR 2	
	Maran et al., 2018^89^	SR/MA	AMSTAR 2	
	Maran et al., 2019^81^	SR/MA	AMSTAR 2	
	Souto-Maior et al., 2018^90^	SR/MA	AMSTAR 2	
8. Can gel agitation improve bleaching efficacy?	Gonçalves et al., 2017^92^	RCT	RoB 2.0	
	Kiyuna et al., 2021^95^	RCT	RoB 2.0	
	Martins et al., 2022^96^	RCT	RoB 2.0	
9. Are over-the-counter products as effective as dentist-supervised bleaching?	Pintado-Palomino et al., 2016^99^	RCT	RoB 2.0	
	Liu, Tu, 2021^100^	RCT	RoB 2.0	
	Ribeiro et al., 2024^101^	RCT	RoB 2.0	
	Ribeiro et al., 2023^102^	RCT	RoB 2.0	
	Collins et al., 2008^103^	RCT	RoB 2.0	
	Tao et al., 2017^104^	RCT	RoB 2.0	
	Zhang et al., 2025^105^	RCT	RoB 2.0	
	Schlafer et al., 2021^106^	RCT	RoB 2.0	
	Meireles et al., 2021^107^	RCT	RoB 2.0	
	Llena et al., 2016^109^	RCT	RoB 2.0	
	Vladislavic et al., 2022^110^	RCT	RoB 2.0	
	Cui et al., 2025^111^	RCT	RoB 2.0	
	Kim et al., 2020^113^	RCT	RoB 2.0	
	Jiang et al., 2019^114^	RCT	RoB 2.0	
	Cordeiro et al., 2019^116^	RCT	RoB 2.0	
	Monteiro et al., 2019^117^	RCT	RoB 2.0	
	Krayem et al., 2024^119^	RCT	RoB 2.0	
	Kim et al., 2018^120^	RCT	RoB 2.0	
	Mailart et al., 2025^121^	RCT	RoB 2.0	
	Oliveira et al., 2024^97^	SR/NMA	AMSTAR 2	
	Rosa et al., 2020^115^	SR/MA	AMSTAR 2	
10. Can desensitizing toothpastes reduce bleaching-induced tooth sensitivity?	Pintado-Palomino et al., 2015^122^	RCT	RoB 2.0	
	Américo et al., 2023^123^	RCT	RoB 2.0	
	Thiesen et al., 2013^126^	RCT	RoB 2.0	
	Ortega-Moncayo et al., 2022^127^	RCT	RoB 2.0	
	Silva et al., 2018^128^	RCT	RoB 2.0	
	Bizreh, Milly, 2022^129^	RCT	RoB 2.0	
	Cabral et al., 2024^124^	SR/MA	AMSTAR 2	

Abbreviations: #: number; RCT: randomized controlled trial; Non-RCT: non-randomized clinical trial; SR: systematic review; MA: meta-analysis; NMA: network meta-analysis; RoB 2.0: Cochrane Risk of Bias tool 2.0; ROBINS-I: Risk Of Bias In Non-randomized Studies of Interventions; AMSTAR 2: A MeaSurement Tool to Assess systematic Reviews. Low risk of bias/low methodological concern;some concerns; high risk of bias/high methodological concern. For systematic reviews, the term "low concern" was used to harmonize the AMSTAR 2 judgment with the terminology used for risk-of-bias tools in this structured narrative synthesis.

Conversely, conclusions were interpreted with greater caution when the evidence was more heterogeneous, protocol-dependent, or included mostly studies with unclear/high risk of bias, as observed in the evidence on OTC products (Myth 9), desensitizing toothpastes (Myth 10) or when few studies were available, such as for tray design (Myth 5). More well-designed RCTs on these topics are required.

Overall, the findings indicate that many bleaching myths persist because a biologically plausible rationale, laboratory observation, traditional teaching, commercial appeal, or isolated clinical result is often interpreted as sufficient justification for routine practice. However, evidence-based bleaching should prioritize interventions that demonstrate measurable patient-centered benefit, acceptable safety, and reproducible clinical outcomes. When evidence is uncertain, the most appropriate clinical position is not to intensify the protocol by default, but to individualize treatment according to patient risk, expected benefit, discomfort, cost, adherence, and the availability of better-supported alternatives.

## Limitations

This review has limitations inherent to its narrative design. Although the search strategy, source types, and rationale for evidence selection were made explicit, this article was not designed as a systematic review, scoping review, or meta-analysis. Therefore, a strict search strategy, predefined eligibility criteria, duplicate study selection, formal data extraction, and quantitative synthesis were not applied and may have missed some articles.

The myths addressed were arbitrarily selected because they represent recommendations frequently asked about in university classes, lectures and bleaching practice, but other relevant clinical questions were not covered in the current literature review. In addition, the amount and quality of evidence varied across topics, with some conclusions supported by consistent RCTs and systematic reviews and others based on more heterogeneous, protocol-dependent, or limited evidence. For this reason, the conclusions should be interpreted as a critical synthesis of the available clinical evidence and not as definitive clinical guidelines. Future well-designed clinical trials and updated systematic reviews may strengthen, refine, or modify some of the interpretations presented here.^1^,^130^

## Conclusions

The clinical myths addressed in this structured narrative review show a common pattern: many widely disseminated practices are supported by biological plausibility, laboratory findings, tradition, or marketing appeal, but do not provide consistent, clinically meaningful benefits when tested in controlled clinical studies. The available evidence indicates that intensifying bleaching protocols by adding light, increasing gel volume or treated surface area, mechanically agitating the gel, unnecessarily prolonging exposure time, or routinely prescribing preventive medication does not usually improve final whitening outcomes and may increase cost, complexity, patient burden, or adverse effects.

Evidence-based dental bleaching should therefore prioritize protocols with demonstrated clinical benefit, acceptable safety, and patient-centered outcomes. Clinical decisions should be guided by the balance between efficacy, TS, GI, treatment time, patient comfort, and professional supervision, rather than by tradition, intuition, isolated findings, or commercial influence.

## Data Availability

The authors declare that all data generated or analyzed during this study are included in this published article.
